# Nasal and Sinonasal Findings in Behçet’s Syndrome: A Scoping Review

**DOI:** 10.3390/jcm15166334

**Published:** 2026-08-16

**Authors:** Yuki Otsuka, Yohei Masuda, Yoshito Nishimura, Yu Katayama, Fumio Otsuka

**Affiliations:** 1 Department of General Medicine, Graduate School of Medicine, Dentistry and Pharmaceutical Sciences, Okayama University, Okayama 700-8558, Japan; otsuka@s.okayama-u.ac.jp (Y.O.); nishimura.yoshito@mayo.edu (Y.N.); fumiotsu@md.okayama-u.ac.jp (F.O.); 2 Division of Hematology/Oncology, Mayo Clinic, Rochester, MN 55905, USA; 3 Department of Rheumatology, Okayama City Hospital, Okayama 700-8557, Japan; yu_katayama@okayama-gmc.or.jp

**Keywords:** Behçet’s syndrome, nose diseases, nasal mucosa, olfaction disorders, mucociliary clearance, otolaryngology, oral ulcer, vasculitis

## Abstract

**Background**: Nasal and sinonasal findings have been reported in patients with Behçet’s syndrome, but their frequency, clinical significance, and relationship to underlying disease remain unclear. We performed a scoping review to map the published literature on epidemiology, clinical characteristics, and management. **Methods**: Following PRISMA-ScR guidance, we systematically searched MEDLINE, EMBASE, and Web of Science from inception through June 2026 for peer-reviewed primary studies reporting nasal or sinonasal findings in patients with Behçet’s syndrome. Two reviewers independently screened studies and extracted data. **Results**: Fifteen eligible studies, including seven case reports and eight observational studies, were identified. Reported frequencies of nasal or sinonasal findings varied substantially across studies. A spectrum of nasal and sinonasal symptoms was noted among the literature, including direct mucosal lesions, sinonasal symptoms and functional abnormalities such as olfactory impairment or delayed mucociliary clearance, and secondary nasal conditions occurring in patients with Behçet’s syndrome. Oral ulcers were consistently reported in the limited studies describing associated systemic features. Treatment data were sparse, and no evidence-based management approach specific to nasal disease was identified. **Conclusions**: The published literature suggests that a range of nasal and sinonasal findings may occur in patients with Behçet’s syndrome. However, the available evidence is sparse, heterogeneous, and insufficient to determine true prevalence, attribution, or optimal management. Future studies should distinguish direct Behçet-related nasal lesions from secondary or nonspecific findings and use standardized clinical assessment.

## 1. Introduction

Behçet’s syndrome is a chronic multisystem inflammatory disorder characterized by recurrent mucocutaneous and systemic manifestations, and is considered to involve both autoinflammatory and autoimmune features [1,2]. It is also classified as a variable-vessel vasculitis that can affect arteries and veins of different calibers [3]. The clinical presentation is heterogeneous, with recurrent oral ulcers, genital ulcers, ocular involvement, skin lesions, and other organ manifestations that make up the disease spectrum [4].

**Figure 1:**
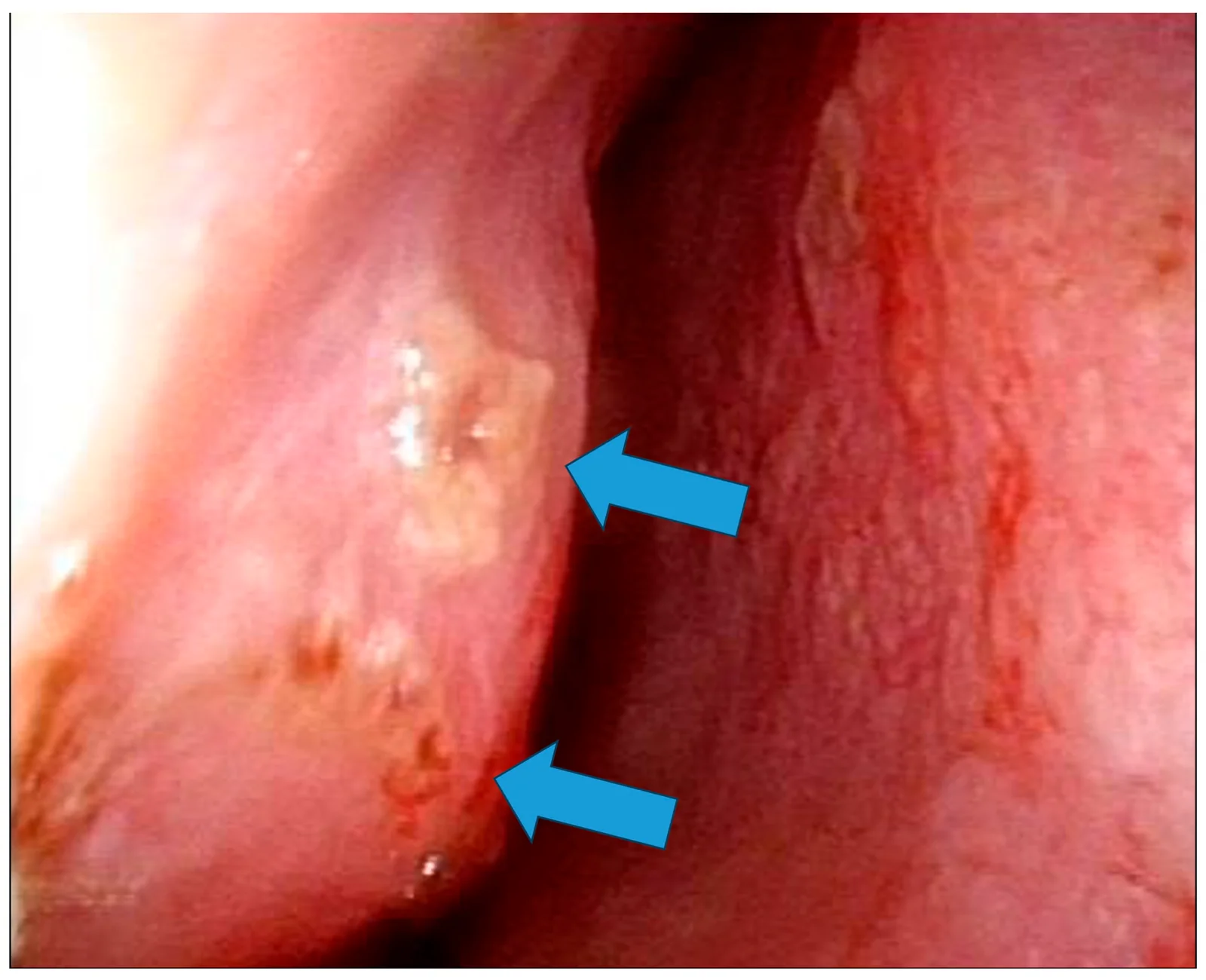
Endoscopic view of nasal vestibule ulcers (blue arrow) in a patient with Behçet’s syndrome. (Akiyama et al. [6], Licensed under CC BY 4.0. https://creativecommons.org/licenses/by/4.0/ (accessed on 18 May 2026)).

Nasal and sinonasal findings, including nasal obstruction, mucosal ulceration, olfactory disturbance, and impaired mucociliary function, have been described in patients with Behçet’s syndrome. However, their clinical significance and causal relationship to the disease remain unclear [5,6] (Figure 1). Nasal symptoms may also occur in relevant clinical contexts such as Mouth and Genital Ulcers with Inflamed Cartilage (MAGIC) syndrome, an overlap phenotype involving Behçet’s syndrome and relapsing polychondritis, although nasal findings are not included in current diagnostic criteria for Behçet’s syndrome itself [7,8].

While prior literature has described selected aspects of nasal involvement in Behçet’s syndrome, the available literature remains scarce and fragmented without structured synthesis that clearly distinguishes primary Behçet-related nasal lesions from nonspecific nasal dysfunction or findings attributable to comorbid conditions. Therefore, we conducted a scoping review to map the published literature on nasal and sinonasal findings reported in patients with Behçet’s syndrome, summarize the available evidence regarding epidemiology, clinical characteristics, and management, and identify key gaps for future research.

## 2. Methods

### 2.1. Study Design

The study was conducted according to the guidelines for scoping reviews [9,10,11]. The findings are reported following the Preferred Reporting Items for Systematic reviews and Meta-Analyses extension for Scoping Reviews (PRISMA-ScR) checklist [12]. PRISMA 2020 checklists are provided in Supplementary Files S1 and S2. The review protocol was not registered, and a separate review protocol was not prepared.

### 2.2. Eligibility Criteria

Eligible studies included peer-reviewed primary reports published in English that described nasal or sinonasal findings in patients with Behçet’s syndrome, including Behçet-related overlap disorders when sinonasal manifestations were reported and were considered clinically relevant to the differential diagnosis of sinonasal manifestations in patients with Behçet’s syndrome. Findings of interest included direct nasal mucosal lesions, sinonasal inflammatory conditions, and nasal functional abnormalities such as olfactory dysfunction or impaired mucociliary clearance that were reported in relation to Behçet’s syndrome. We excluded review articles, editorials, commentaries, conference abstracts without full reports, and studies in which Behçet’s syndrome or a Behçet-related overlap disorder was not clearly identified.

### 2.3. Search Strategy

A search of the MEDLINE, EMBASE, and Web of Science databases was conducted for all peer-reviewed articles published from their inception through June 2026. The search strategy combined terms related to Behçet’s syndrome with nasal and sinonasal manifestations, and the complete search strategies for each database are provided in Appendix A. The initial search was performed without language restrictions. Only English-language full-text studies were included at the eligibility stage. Additionally, the reference lists of all eligible articles were manually screened to identify further relevant studies. Two authors (YN and YO) conducted the search independently. The final search results were exported to EndNote 21 [13], where duplicates were removed by one author (YM). The cleaned references were then imported into the Rayyan software [14] to facilitate the screening process.

### 2.4. Article Selection

Two authors (YM and YO) independently screened the titles and abstracts of all identified articles. The full texts of potentially relevant studies were then retrieved and assessed against the eligibility criteria for final inclusion. Any disagreements between the authors regarding article eligibility were resolved through discussion until a consensus was reached.

### 2.5. Data Extraction

A standardized data-charting form was developed based on the PRISMA-ScR guidance and piloted prior to actual data extraction. Two reviewers independently charted key study characteristics, including author, year, country, study design, sample size, patient demographics, Behçet-related clinical characteristics, type of nasal or sinonasal finding, endoscopic or imaging findings, functional assessments, histopathologic information when available, treatment details, and authors’ interpretation of attribution to Behçet’s syndrome.

### 2.6. Data Synthesis

A descriptive qualitative synthesis was performed. Findings were summarized descriptively according to study design and reported clinical characteristics. We also summarized available data on associated systemic manifestations, endoscopic or imaging findings, functional testing, and treatment approaches.

## 3. Results

### 3.1. Search Results and Characteristics of Included Articles

**Figure 2:**
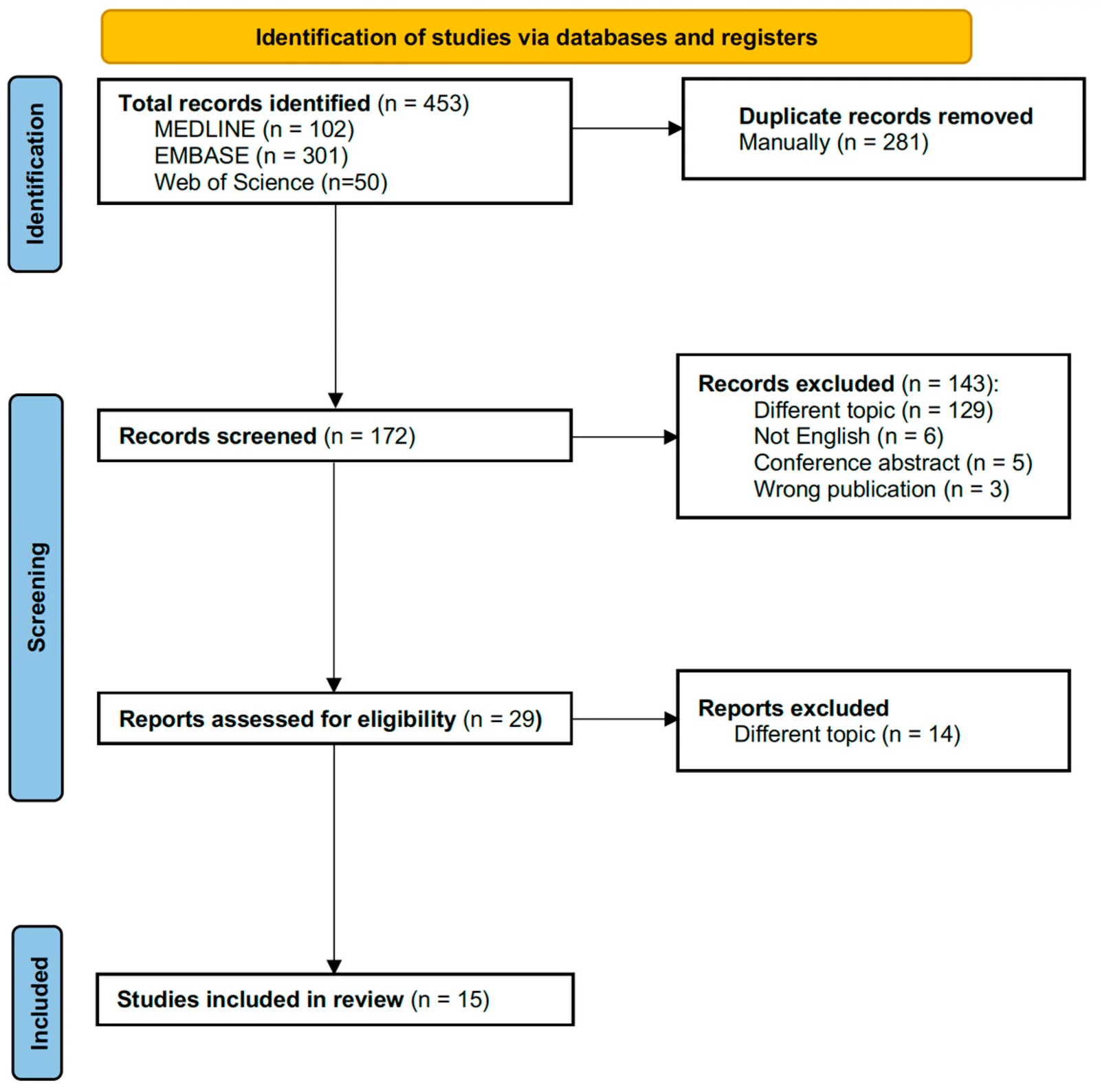
PRISMA 2020 flow diagram for the selection of studies in this study [28]. Licensed under CC BY 4.0. https://creativecommons.org/licenses/by/4.0/ (accessed on 18 May 2026).

Figure 2 illustrates the PRISMA flow diagram for the article selection process. The initial search yielded 453 articles. After 281 duplicates were removed, the titles and abstracts of the remaining 172 articles were screened, leading to the exclusion of clearly ineligible articles. A total of 29 articles underwent full-text screening, and ultimately, 15 articles were included in this review [5,6,15,16,17,18,19,20,21,22,23,24,25,26,27]. The 15 included articles consisted of seven case reports and eight original articles. They were clinically and methodologically heterogeneous, including direct nasal mucosal lesions, cohort studies of sinonasal symptoms or dysfunction in patients with Behçet’s syndrome, and studies describing nasal findings in related or overlapping clinical contexts.

### 3.2. Epidemiology and Risk Factors

While epidemiological data on nasal involvement in Behçet’s syndrome are limited, a report from Iran noted that among 400 patients with the disease, 67 (17%) had a history of nasal symptoms, most commonly including ulceration, pain, a burning sensation, and nasal obstruction [5]. They also reported that 31 patients (7.8%) experienced ongoing symptoms, including olfactory disturbance and nasal obstruction. In contrast, a cohort study from Mongolia reported that nasal mucosal ulcers were present in 36 of 65 patients (55.4%), including 14 males and 22 females [23]. These findings suggest considerable variation in the prevalence of nasal involvement in Behçet’s syndrome. Climatic and geographical factors, such as temperature fluctuations and desert environments, were proposed by the authors as possible explanations for this variation [23]. However, this difference should be interpreted with caution, as variations in case definitions, patient selection, referral settings, diagnostic criteria, and disease severity may also have contributed to the observed differences in prevalence. Only one study described potential risk factors for nasal involvement in Behçet’s syndrome, which indicated that the duration of the disease was significantly correlated with impaired nasal mucociliary function [22].

### 3.3. Clinical Presentations

Details of the clinical presentations of the included cases are summarized in Table 1 [6,15,16,17,18,24,27]. Among the seven case reports, the reported nasal presentations included nasal obstruction, pain, epistaxis, rhinosinusitis, and ulcerative or vesicular lesions. Attribution to Behçet’s syndrome varied across cases. Four reports were classified as primary Behçet-related nasal manifestations, including Behçet-related nasal ulceration, postoperative pathergy-related lesions, and rhinosinusitis attributed to Behçet-related vasculitis [6,16,18,27]. The remaining three reports described associated conditions occurring in patients with Behçet’s syndrome, including malignant lymphoma, nasal endometriosis, and intranasal herpes simplex virus infection [15,17,24]. Observational otolaryngology-based studies described a broader range of sinonasal presentations, including acute and chronic rhinosinusitis. In one study, chronic rhinosinusitis was reported more frequently in patients with Behçet’s syndrome than in controls [20]. A separate study of MAGIC syndrome also reported nasal chondritis [25].

### 3.4. Laboratory Findings

The included literature did not allow robust assessment of whether nasal or sinonasal findings may be associated with specific Behçet phenotypes or laboratory findings. HLA data were only available in one study that reported HLA-B51 positivity in 31% of patients with nasal symptoms, but no included study evaluated whether HLA-B51 was associated with a higher likelihood of nasal involvement relative to Behçet patients without such findings [5].

### 3.5. Endoscopic and Functional Findings

Three observational studies compared nasal function between patients with Behçet’s syndrome and controls, evaluating olfactory performance, mucociliary clearance, and sinonasal disease severity [20,21,22]. Overall, the studies reported possible functional impairment in some domains, although the findings were not uniform (Table 2). For example, odor identification and total olfactory scores were lower in one Behçet cohort, whereas odor discrimination was not significantly different. Another study found prolonged mucociliary clearance time in Behçet’s syndrome. In contrast, objective sinonasal severity measures were not consistent among the cases. Endoscopic assessment was reported in several studies, but symptoms did not correlate with the endoscopic findings [17,18,19,20,23,24,26].

Patients with positive endoscopic findings more often reported nasal obstruction, rhinorrhea, or pain, yet some symptomatic patients had no clear endoscopic abnormalities. The endoscopic pattern was heterogeneous without consistent findings across the literature. and not uniformly supported across all objective measures.

### 3.6. Associated Systemic Manifestations

The frequencies of associated manifestations, including skin, ocular, genital, articular, vascular, and neurological involvement, are summarized in Table 3. The International Study Group (ISG) criteria for Behçet’s syndrome were explicitly reported in five studies [19,20,22,23,26], whereas the remaining studies either used other diagnostic criteria or did not specify the criteria used. Three studies described the accompanying clinical features in patients with Behçet’s syndrome who presented with nasal symptoms [5,19,21]. All studies consistently reported oral ulcers.

### 3.7. Treatment Approaches

Evidence regarding treatment was limited. Among case reports, localized approaches including nasal irrigation, topical antibiotics, Gelfoam application, and placement of a septal button were not successful in the single detailed case of a Behçet-related nasal ulcer, in which systemic colchicine was ultimately required [18]. This case suggests that reconstructive interventions may be considered in patients who develop severe tissue destruction after adequate control of the underlying inflammatory disease. However, evidence regarding the indications, timing, and outcomes of reconstructive surgery in Behçet’s syndrome remains extremely limited. When nasal findings were secondary to other conditions, management was directed primarily at the alternative diagnosis, such as chemotherapy for lymphoma, antiviral therapy for herpes simplex (HSV) virus infection, or hormonal therapy for endometriosis [15,17,24]. None of the observational studies provided sufficient treatment detail to support recommendations regarding optimal management of nasal or sinonasal findings in Behçet’s syndrome.

## 4. Discussion

In this scoping review, we found that nasal and sinonasal findings have been reported in patients with Behçet’s syndrome across a small and heterogeneous body of literature. The available evidence includes direct mucosal lesions, broader sinonasal symptoms, and functional abnormalities, as well as secondary nasal conditions occurring in patients with Behçet’s syndrome. Although these reports suggest that nasal or sinonasal involvement may be clinically relevant in a subset of patients, the current evidence is insufficient to define its true prevalence, establish causality for the reported findings, or support its consideration as a defining feature of Behçet’s syndrome. Further research in the area is required to confirm its real-world prevalence and its burden for the patients.

The heterogeneity of the included reports suggests that nasal involvement in Behçet’s syndrome is unlikely to represent a single clinicopathologic entity. Some reports described ulcerative lesions or pathergy-compatible phenomena that may plausibly reflect Behçet-related mucosal inflammation, whereas others described nonspecific dysfunction or nasal pathology more likely attributable to comorbid conditions. This heterogeneity among the literature limits the current diagnostic utility of nasal findings and argues against treating all reported nasal abnormalities in the patients as biologically equivalent. The heterogeneity of the functional studies should also be considered when interpreting these findings. The included studies evaluated different aspects of sinonasal function using distinct assessment methods, including olfactory testing and mucociliary clearance, and therefore are not directly comparable. Differences in study populations and control selection further limit interpretation of these findings.

Similarly, although one study reported HLA-B51 positivity in 31% of patients with nasal symptoms [5], no study compared HLA-B51 frequency between patients with and without sinonasal involvement. Therefore, the clinical significance of HLA-B51 in relation to nasal manifestations remains uncertain. Likewise, the marked differences in the reported prevalence of nasal ulceration across studies should be interpreted cautiously. In addition to possible climatic or geographical influences [23], variations in study populations, disease duration, diagnostic criteria, methods of nasal assessment, and underlying genetic backgrounds may also have contributed to these discrepancies.

In the meantime, nasal manifestations in patients with Behçet’s syndrome may be underrecognized, but they may also be misattributed to Behçet’s syndrome. Among the published cases, not all sinonasal lesions represented primary manifestations of Behçet’s syndrome. Important differential diagnoses include common sinonasal disorders such as rhinitis and chronic rhinosinusitis, as well as inflammatory diseases such as granulomatosis with polyangiitis, eosinophilic granulomatosis with polyangiitis, relapsing polychondritis, IgG4-related disease, sarcoidosis, and Crohn disease, as well as extranodal NK/T-cell lymphoma, chronic infections, and cocaine induced midline destructive lesions [29,30]. Differentiation from granulomatosis with polyangiitis is particularly important because destructive sinonasal disease is a hallmark of granulomatosis with polyangiitis but is uncommon in Behçet’s syndrome. In addition, several included case reports highlighted alternative diagnoses or overlap disorders that should be considered in the differential diagnosis of sinonasal manifestations in patients with Behçet’s syndrome, including malignant lymphoma, nasal endometriosis, postoperative pathergy-related lesions, intranasal HSV infection, and a Behçet-related overlap disorder. Prematurely attributing sinonasal findings to Behçet’s syndrome may result in diagnostic error through cognitive biases such as anchoring bias or premature closure. Careful clinical evaluation, including appropriate microbiological, radiological, or histopathological assessment when indicated, is therefore essential before concluding that sinonasal manifestations are directly related to Behçet’s syndrome.

Because direct treatment evidence is scarce, management of suspected Behçet-related nasal ulceration may be extrapolated from principles used for non-nasal mucocutaneous Behçet disease. While supportive localized measures, including humidification and avoidance of irritative exposures, may help symptom control, the limited published reports suggest that systemic therapy such as colchicine may be required in selected cases. For patients with chronic rhinosinusitis or severe structural complications, surgical intervention may occasionally be considered after appropriate medical treatment and adequate control of the underlying inflammatory disease. However, no study included in this review evaluated the indications, timing, or outcomes of sinonasal surgery in Behçet’s syndrome. Although endoscopic sinus surgery has been described for other complex inflammatory sinonasal conditions [31], its role in Behçet’s syndrome remains undefined. The role of topical intranasal therapy appears uncertain and may be primarily adjunctive, although further research is required to quantify its efficacy in controlling the disease.

The review has several limitations. First, the available evidence base was small and heterogeneous, consisting largely of case reports and small observational studies. The functional studies also used different outcome measures and assessment methods, limiting cross-study comparison and increasing the risk of selective reporting bias. Second, reporting and referral biases are likely, particularly because patients with more severe or unusual nasal symptoms may be more likely to undergo otolaryngologic evaluation and publication. In addition, most studies originated from a limited number of countries, and genetic data, including HLA-B51, were scarce, limiting the generalizability of the findings. Third, the reviewed literature did not consistently distinguish primary Behçet-related lesions from secondary nasal conditions or nonspecific functional abnormalities, which limits causal interpretation. Finally, because treatment data were sparse and comparative studies were lacking, rigorous management strategies cannot be derived from the current literature.

## 5. Conclusions

In summary, the available literature indicates that a range of nasal and sinonasal findings can occur in patients with Behçet’s syndrome. However, the evidence is limited to determine their true prevalence, define a distinct nasal phenotype, or establish optimal management. Future studies should distinguish direct Behçet-related nasal lesions from secondary or nonspecific findings and should incorporate standardized clinical, endoscopic, and pathologic assessment. Multidisciplinary collaboration between rheumatologists and otolaryngologists may be crucial to clarify the significance of these findings in clinical practice.

## Figures and Tables

**Table 1:** Clinical characteristics of seven case reports.

Author, Year, Country	Age, Sex	Presumed Etiology	Symptoms	Treatment for Nasal Lesion	Behçet’s Syndrome-Associated Findings/Comorbidities
Primary Behçet-related nasal manifestations					
Sen, 2004, [16] Turley.	38, M	Aphthous pustular lesions after surgery	Dampness underneath the splint	Exposed and frequently applied ointment	Oral lesions, Skin lesions, Genital ulcer, Arthritis
Alcântara, 2005, [27] Brazil.	11, F	Rhinosinusitis (periorbitary abscess)	Facial pain, purulent rhinorrhea, nasal obstruction, headache	(1) Ceftriaxone, (2) Orbital abscess incision and drainage, (3) Penrose drain placement, (4) Nasal debridement	Oral lesions, Ocular lesions, Genital ulcer, convulsions
Meyer, 2014, [18] USA.	14, F	Ulcer due to Behçet’s syndrome itself	Epistaxis	(1) Nasal irrigation and topical antibiotics, (2) Gelfoam, (3) Septal button, (4) Colchicine	Oral lesions, Ocular lesions, Arthritis, Nodular acne vulgaris, Axillary hyperhidrosis, Attention deficit hyperactivity disorder
Akiyama, 2025, [6] Japan.	25, M	Ulcer due to Behçet’s syndrome itself	Rhinalgia, nasal obstruction	(1) Colchicine, (2) Apremilast, (3) Prednisolone	Oral lesions, Skin lesions, Genital ulcer, Intestinal lesions,
Associated conditions occurring in patients with Behçet’s syndrome					
Kaneko, 1974, [15] Japan.	32, M	Nasal malignant lymphoma, gangrenous rhinitis	Rhinostenosis	Steroid, Indomethacin, 60 Co irradiation, Vincristine, Cyclophosphamide, 6-mercaptopurine, Bleomycin	Oral lesions, Ocular lesions, Arthritis, Intestinal lesions, Myocarditis, Rhinitis hypertrophicans, Gangrenous rhinitis
Mignemi, 2012, [17] Italy.	35, F	Nasal endometriosis	Epistaxis and nasal pain	(1) Discontinuation of combined oral contraceptive, (2) Progestin, (3) Gonadotropin-releasing hormone analog	Skin lesions, Ocular lesions, Intestinal lesions, Endometriosis
Tsunoda, 2020, [24] Japan.	52, F	Blisters due to intranasal HSV infection	Left nostril pain	(1) Vidarabine ointment, (2) oral famciclovir	Oral lesions, Intestinal lesions, Vascular lesions

**Table 2:** Endoscopic and functional findings among Behçet’s syndrome patients.

Author, Year, Country	Tests/Scores	Behçet’s Syndrome	Non-Behçet’s Syndrome	*p* Value
Verim, 2015, [20] Turkey.	Lund-Mackay Endoscopy Score (Left)	1.64 (SD 1.283)	1.18 (SD 0.984)	*p* = 0.023 *
	Lund-Mackay Endoscopy Score (Right)	1.46 (SD 1.267)	1.08 (SD 1.017)	*p* = 0.061
Akyol, 2016, [21] Turkey.	Odor identification score (Mean ± SD)	3.2 ± 1.7	6.2 ± 2.1	*p* < 0.001 *
	Odor discrimination score (Mean ± SD)	8.8 ± 2.1	9.4 ± 1.6	*p* = 0.15
	Total Score (Sniffin’ Sticks olfactory test) (Mean ± SD)	12 ± 3.4	15 ± 3.1	*p* < 0.001 *
Ozbay, 2016, [22] Turkey.	Nasal mucociliary clearance time (Mean ± SD)	13.4 ± 3.3	9.0 ± 1.8	*p* < 0.001 *

* *p* < 0.05, SD; Standard Deviation.

**Table 3:** Associated manifestations of Behçet’s syndrome to nasal presentation.

Author, Year, Country	Oral Aphthous Ulcers	Skin Lesions	Ocular Lesions	Genital Ulcers	Arthritis	Epididymitis	Intestinal Lesions	Vascular Lesions	Neurological Lesions	Pathergy Test
Shahram, 2010, [5] Iran.	31/31	17/31	18/31	18/31	6/31	1/31	0/31	1/31	5/31	
Veyseller, 2014, [19] Turkey.	30/30	23/30	7/30	15/30						23/30
Akyol, 2016, [21] Turkey.	50/50	48/50	13/50	36/50	15/50			20/50	4/50	

## Data Availability

No new data were created or analyzed in this study. Data sharing is not applicable to this article.

## Supplementary Materials

The following supporting information can be downloaded at: https://www.mdpi.com/article/10.3390/jcm15166334/s1. Supplementary File S1. PRISMA 2020 for Abstracts Checklist. Supplementary File S2. PRISMA 2020 Checklist.

## Abbreviations

The following abbreviations are used in this manuscript:

EMBASE	Excerpta Medica Database
HLA	Human Leukocyte Antigen
HLA-B51	Human Leukocyte Antigen B51
HSV	Herpes Simplex Virus
ISG	International Study Group
MAGIC syndrome	Mouth and Genital Ulcers with Inflamed Cartilage syndrome
MEDLINE	Medical Literature Analysis and Retrieval System Online
PRISMA-ScR	Preferred Reporting Items for Systematic reviews and Meta-Analyses extension for Scoping Reviews

## Appendix A. Search Terms for Each Database

MEDLINE:

(“behcet syndrome”[MeSH Terms] OR “behcet”[All Fields]) AND (“nose”[MeSH Terms] OR “nose”[All Fields] OR “nasal”[All Fields] OR “sinonasal”[All Fields] OR “rhinosinusitis”[MeSH Terms] OR “rhinosinusitis”[All Fields])

EMBASE:

‘Behcet’ AND (‘nose’ OR ‘nasal’ OR ‘sinonasal’ OR ‘rhinosinusitis’)

Web of Science:

TS = (Behcet) AND TS = (nose OR nasal OR sinonasal OR rhinosinusitis)
